# Integrative Analysis Reveals Key Circular RNA in Atrial Fibrillation

**DOI:** 10.3389/fgene.2019.00108

**Published:** 2019-02-19

**Authors:** Xiaofeng Hu, Linhui Chen, Shaohui Wu, Kai Xu, Weifeng Jiang, Mu Qin, Yu Zhang, Xu Liu

**Affiliations:** ^1^Department of Cardiology, Shanghai Chest Hospital, Shanghai Jiao Tong University, Shanghai, China; ^2^Department of Neurology, Zhejiang Hospital, Hangzhou, China

**Keywords:** circular RNAs, atrial fibrillation, ceRNA network, PPI network, inflammatory responses

## Abstract

Circular RNAs (circRNAs) are an emerging class of RNA species that may play a critical regulatory role in gene expression control, which can serve as diagnostic biomarkers for many diseases due to their abundant, stable, and cell- or tissue-specific expression. However, the association between circRNAs and atrial fibrillation (AF) is still not clear. In this study, we used RNA sequencing data to identify and quantify the circRNAs. Differential expression analysis of the circRNAs identified 250 up- and 126 down-regulated circRNAs in AF subjects compared with healthy donors, respectively. Gene Ontology (GO) and Kyoto Encyclopedia of Genes and Genomes (KEGG) analysis of the parental genes of the dysregulated circRNAs indicated that the up-regulated parental genes may participate in the process of DNA damage under oxidative stress. Furthermore, to annotate the dysregulated circRNAs, we constructed and merged the competing endogenous RNA (ceRNA) network and protein-protein interaction (PPI) network, respectively. In the merged network, 130 of 246 dysregulated circRNAs were successfully characterized by more than one pathway. Notably, the five circRNAs, including chr9:15474007-15490122, chr16:75445723-75448593, hsa_circ_0007256, chr12:56563313-56563992, and hsa_circ_0003533, showed the highest significance by the enrichment analysis, and four of them were enriched in cytokine-cytokine receptor interaction. These dysregulated circRNAs may mainly participate in biological processes of inflammatory response. In conclusion, the present study identified a set of dysregulated circRNAs, and characterized their potential functions, which may be associated with inflammatory responses in AF. To our knowledge, this is the first study to uncover the association between circRNAs and AF, which not only improves our understanding of the roles of circRNAs in AF, but also provides candidates of potentially functional circRNAs for AF researchers.

## Introduction

Atrial fibrillation (AF) is one of the most common arrhythmias, which is closely associated with poor life quality, stroke, heart failure, and elevated mortality (Chu et al., 2013; Lang et al., 2014). The number of individuals with AF worldwide in 2010 was estimated to be about 33.5 million (Chugh et al., 2014). The prevalence of AF varies regionally according to previous reports, ranging from 0.1% in India (Kaushal et al., 1995) to 1–2% in Europe and North America (Go et al., 2001; Krijthe et al., 2013) and 4% in Australia (Middleton et al., 2002). The prevalence and incidence of AF have been reported to be higher in European ancestry than non-Europeans (Go et al., 2001; Ball et al., 2013). The occurrence and development of AF are significantly associated with multiple risk factors, including aging (Chugh et al., 2014), male sex (Ball et al., 2013), ethnicity (Rodriguez et al., 2015), cigarette smoking (Ball et al., 2013), alcohol consumption (Ball et al., 2013), obesity (Rahman et al., 2014), hypertension, left ventricular hypertrophy (LVH), coronary artery disease (CAD) (Schnabel et al., 2009), heart failure (HF) (Wang et al., 2003), and valve disease (Rahman et al., 2014).

With the development of high-throughput technologies, such as microarray, next generation sequencing, and mass-spectrum based proteomics, our understanding of the AF pathogenic mechanisms at different levels has been greatly improved. Previous studies (Uemura et al., 2004; Pei et al., 2010; Li et al., 2011; Yao et al., 2015; Mase et al., 2017) used a variety of means to uncover potential molecules responsible for the pathogenesis of AF. For example, genome-wide association studies (Benjamin et al., 2009; Ellinor et al., 2010, 2012; Sinner et al., 2014; Christophersen et al., 2017; Low et al., 2017) have identified at least 30 loci associated with AF, which expand the diversity of genetic pathways implicated in AF and provide novel molecular targets for future biological investigation. Furthermore, transcriptome analysis is one of the most utilized approaches to study human diseases at the mRNA level (Casamassimi et al., 2017). It has been used to define the atrial mRNA expression in different types of AF (e. g., postoperative, chronic, and paroxysmal) (Kim et al., 2003, 2005; Ohki et al., 2005; Deshmukh et al., 2015). In addition to transcriptome analysis, mass-spectrometry-based proteomics has matured into a broadly applied analytical tool over the past decade (Aebersold and Mann, 2016). Mayr et al. (2008) and Zhang et al. (2013) performed proteome analyses in left and right human atrial appendages with persistent AF and found 17 and 223 differentially expressed proteins compared to patients with sinus rhythm. These studies suggest that the pathogenesis of AF is multifactorial, and highlight the association between increased inflammatory burden and the presence and future development of AF (Kourliouros et al., 2009). However, the increased morbidity of AF suggested that some specific pathogenic mechanisms have not been fully understood.

Recently, there is growing evidence that non-coding RNAs, including microRNAs, small nucleolar RNAs and long non-coding RNAs, play important roles in occurrence and development of diseases (Shi et al., 2013; Ruan et al., 2015; Yi et al., 2018). Furthermore, circular RNAs are emerging as a new type of regulatory molecules that participate in gene expression control and disease progression (Han et al., 2018). In AF, circRNA-associated ceRNA networks revealed that dysregulated circRNAs (hsa_circRNA002085, hsa_circRNA001321) in non-valvular persistent atrial fibrillation (NPAF) may be involved in regulating hsa-microRNA (miR)-208b and hsa-miR-21 (Zhang et al., 2018). Moreover, bioinformatics analysis provides a novel perspective on circRNAs involved in AF due to rheumatic heart disease and established the foundation for future research of the potential roles of circRNAs in AF. To uncover the association between circRNAs and AF, we performed an integrative analysis of circRNAs, and identified dysregulated circRNAs in lymphocytes of AF. The functions of the dysregulated circRNAs were annotated by network-based Kyoto Encyclopedia of Genes and Genomes (KEGG) pathway enrichment analysis, which highlighted several circRNAs participating in biological processes of inflammatory response.

## Materials and Methods

### Data Collection and Format Conversion

RNA sequencing data of 6 cases with AF and 6 healthy donors were downloaded from Sequence Read Archive (SRA)^1^ database (Leinonen et al., 2011) with an accession number SRP093226 using SRA Toolkit (Leinonen et al., 2011) version 2.9.2^2^, which was released by previous study (Yu et al., 2017). The downloaded files with SRA format were converted to paired-end FASTQ files by fastq-dump with the option *–split-files*.

### RNA Sequencing Data Analysis

The RNA sequencing data were analyzed by two pipelines. For the gene expression quantification, we mapped the RNA-seq reads to UCSC human reference genome (hg19)^3^ by samples using hisat2 (Kim et al., 2015). The resulting SAM files were sorted by SAMtools. Gene expression was quantified by StringTie (Pertea et al., 2015) with GENCODE (Harrow et al., 2012) annotation v19. For the circular RNA detection and quantification, we used the BWA-mem aligner to map the RNA-seq reads to UCSC human reference genome (hg19). The circular RNAs were predicted and quantified by CIRI-2 with GENCODE (Harrow et al., 2012) annotation v19.

### Identification of Highly Reliable Circular RNAs Using RNA-seq Data

To identify the circular RNAs, we filtered the circRNAs with more than 5-read counts in more than two samples. Moreover, the threshold of the average ratio of junction reads supporting circRNAs was also set to 10%.

### Differential Expression Analysis

The count-based expression was used to identify differentially expression genes and circRNAs by DESeq2 (Love et al., 2014), a differential expression analysis based on the negative binomial distribution. The gene and circRNA expression were normalized to avoid the influence of sequencing depth and transcript length, and was implemented in R package DESeq2. The differentially expressed genes/circRNAs were identified at the threshold *P*-value < 0.05 and fold change > 2 or < 1/2. The up- or down-regulation status was determined based on the fold change for each gene/circRNA.

### GO and KEGG Enrichment Analysis

The Gene Ontology (GO) and KEGG enrichment analysis was implemented at WEB-based Gene Set Analysis Toolkit (WebGestalt) (Wang et al., 2017). The Gene Ontology (Ashburner et al., 2000) biological processes and KEGG pathways (Kanehisa et al., 2017) were selected as the functional database.

### Protein-Protein Interaction Analysis

The Search Tool for the Retrieval of Interacting Genes/ Proteins (STRING) (Szklarczyk et al., 2017) online software^4^ was used to assess the interactions. The interactions of the proteins encoded by the differently expressed genes were searched using STRING online software.

### MiRNA Target Prediction

The miRNA binding sites of circRNAs were predicted by Miranda (Betel et al., 2008) with option *–strict*. We selected default options for other parameters. The miRNA-mRNA interactions were extracted from MiRTarBase (Chou et al., 2018). Together with the reverse co-expression analysis of miRNA and mRNA, miRNA and mRNA interaction pairs were predicted.

### Competing Endogenous RNA Prediction

The competing endogenous RNAs (ceRNAs) function by competing for miRNAs with mRNAs. The number of miRNAs shared by each circRNA and mRNA pair should be significantly higher. For each mRNA-circRNA pair, Fisher’s exact test was used to estimate the significance of shared miRNAs (*P*-value < 0.0001).

### Functional Annotation of circRNAs

The protein-protein interaction (PPI) and ceRNA network were merged and visualized using Cytoscape software^5^. The function of circRNAs were predicted by the KEGG pathway (Kanehisa et al., 2017) enrichment analysis performed on the genes connected to these circRNAs within one node in the merged network.

### Statistical Analysis

The statistical analyses, such as hierarchical clustering analysis and Fisher’s exact test, were implemented in R programming software^6^.

## Results

### Identification of circRNAs in Lymphocytes From Atrial Fibrillation and Healthy Donors

**FIGURE 1:**
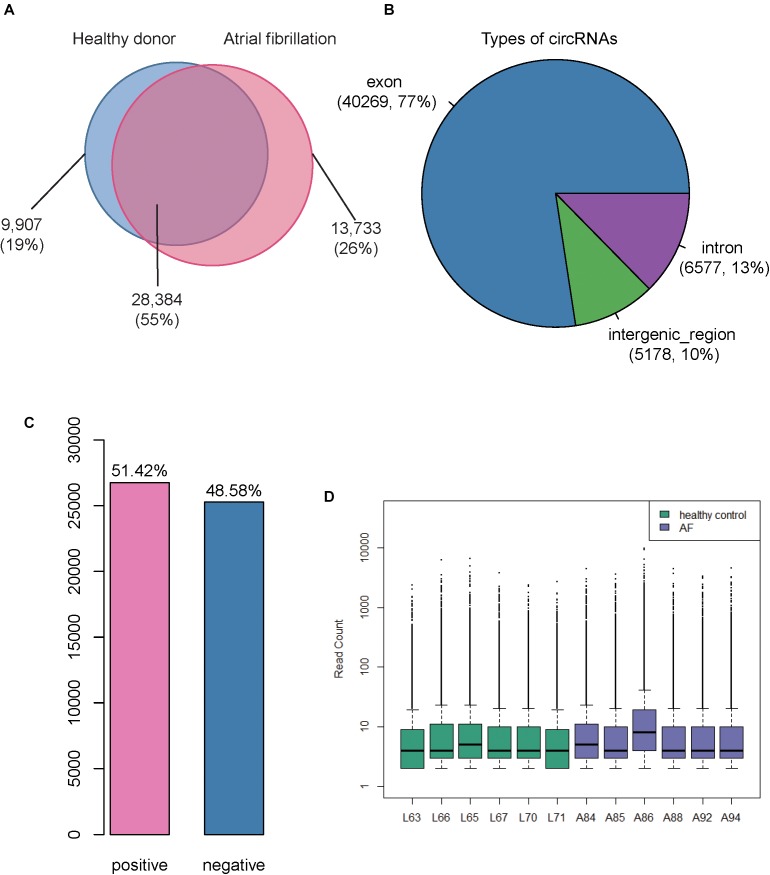
Overview of the identified Circular RNAs (circRNAs) in atrial fibrillation (AF) and healthy controls. **(A)** The Venn diagram displays the number of circRNAs identified in AF and healthy controls, respectively. **(B)** The pie chart displays the ratio and number of circRNAs originated from exonic, intronic, and intergenic regions. **(C)** The number and ratio of circRNAs transcribed from sense and antisense strands. **(D)** The distribution of count-based circRNA expression in each sample. The purple and green boxes represent the AF and healthy control samples, respectively.

We collected RNA sequencing data of 6 cases with atrial fibrillation and 6 healthy donors from SRA^7^ database with an accession number SRP093226 (Yu et al., 2017) (see section “Materials and Methods”), the RNA libraries of which were constructed by rRNA-removal protocol and could be used to identify circular RNAs (circRNAs). As described in the previous study, two and three male samples were collected in AF and healthy controls, respectively. Moreover, all samples did not have smoking habits and alcohol abuse. Particularly, the average age of AF patients was about 62 years old, which was slightly older than that of healthy controls. The analysis of sequencing data allowed for identifying 52,024 circRNAs in total, of which, 28,384 were identified in both atrial fibrillation and healthy donors (Figure 1A). Among these identified circRNAs, we observed that 13,899 were curated by a circRNA database, circBase^8^ (Glazar et al., 2014). Moreover, we also found 13,733 and 9,907 circRNAs to be specific to the atrial fibrillation and healthy donors, respectively (Figure 1A). Genomic annotations revealed that these circRNAs were mostly originated from the exons (77%), followed by introns (13%) and intergenic regions (10%), suggesting that a considerable portion of circRNAs were circularized at unannotated splicing sites in lymphocytes (Figure 1B). The ratio of circRNAs transcribed from the sense strands was close to 0.5, indicating that there was not strand-preference in circRNA biogenesis (Figure 1C). In addition, we also examined the distribution of circRNAs expression levels in each sample, and observed that most of circRNAs were expressed at low levels (Figure 1D). However, there were also about 25% circRNAs in each sample that were expressed at a higher level (> 30 read count, Figure 1D).

### Identification of Dysregulated Genes and circRNAs in Atrial Fibrillation

**FIGURE 2:**
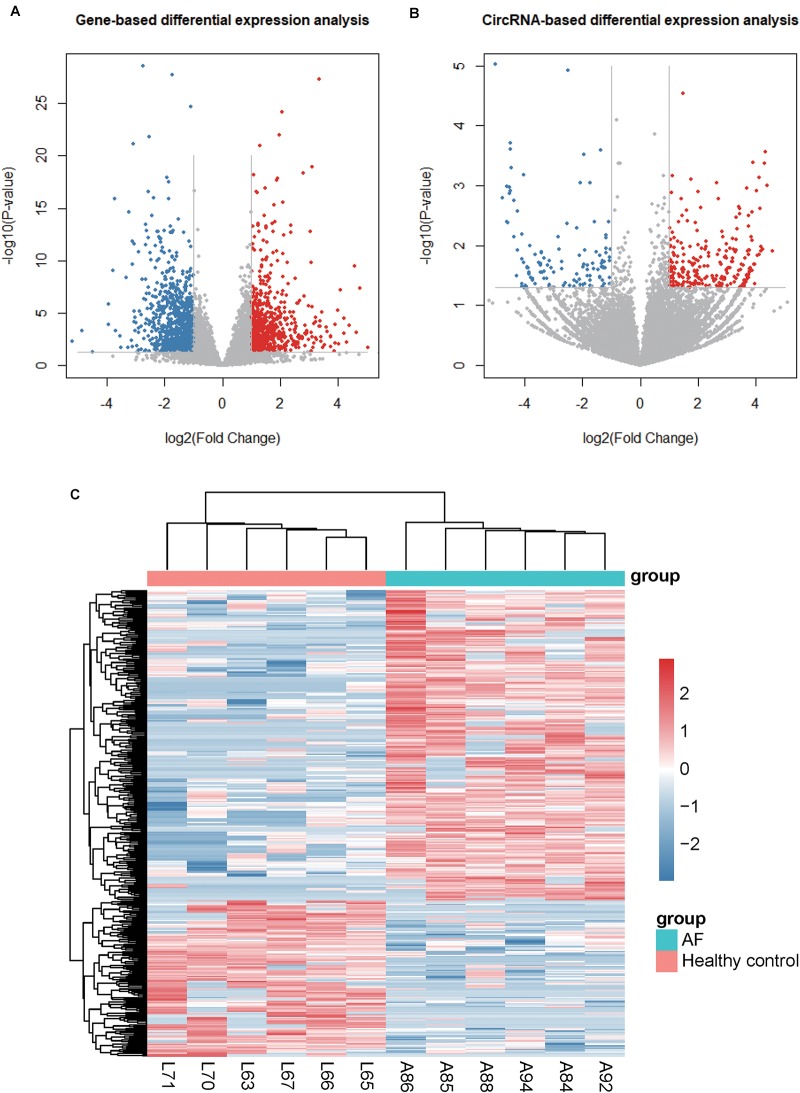
Differentially expressed genes and circRNAs. **(A)** and **(B)** display the volcano plots for gene- and circRNA-based differential expression analysis, respectively. **(C)** The dysregulated circRNAs and samples are co-clustered by hierarchical clustering analysis. The count-based expression levels are normalized, log2-transformed and scaled by circRNA.

To identify the dysregulated genes and circRNAs, we performed differential expression analysis on the gene and circRNA expression profiles, respectively. We identified 713 up- and 994 down-regualated genes, and 250 up- and 126 down-regulated circRNAs in atrial fibrillation compared with healthy donors (*P* < 0.05 and fold change > 2 or < ^1^/_2_, Figure 2A,B), respectively. The hierarchical clustering analysis of the dysregulated circRNA expression profiles revealed that the samples with AF could be clearly distinguished from the healthy donors (Figure 2C), suggesting that the dysregulated circRNAs may act as potential AF diagnostic biomarkers in lymphocytes. Notably, we observed an up-regulated circRNA, hsa_circ_0030569, in AF patients (*P*-value < 0.05 and fold change > 1), which has been reported to response to Mycobacterium tuberculosis (Mtb) infection in human monocyte derived macrophages (MDMs), suggesting that this circRNA may participate in immune or inflammatory processes (Huang et al., 2017).

### GO and KEGG Analysis of the Dysregulated circRNA Parental Genes

It has been shown in previous studies that there is a close association between circRNAs and their parental genes as they could affect the expression of their parental genes (Zhang et al., 2016; Wei et al., 2017). To investigate the functions of the parental genes of dysregulated circRNAs in AF samples compared with normal samples, we conducted a gene set enrichment analysis of their parental genes based on biological processes from GO and pathways from KEGG database (Supplementary Table S1).

**FIGURE 3:**
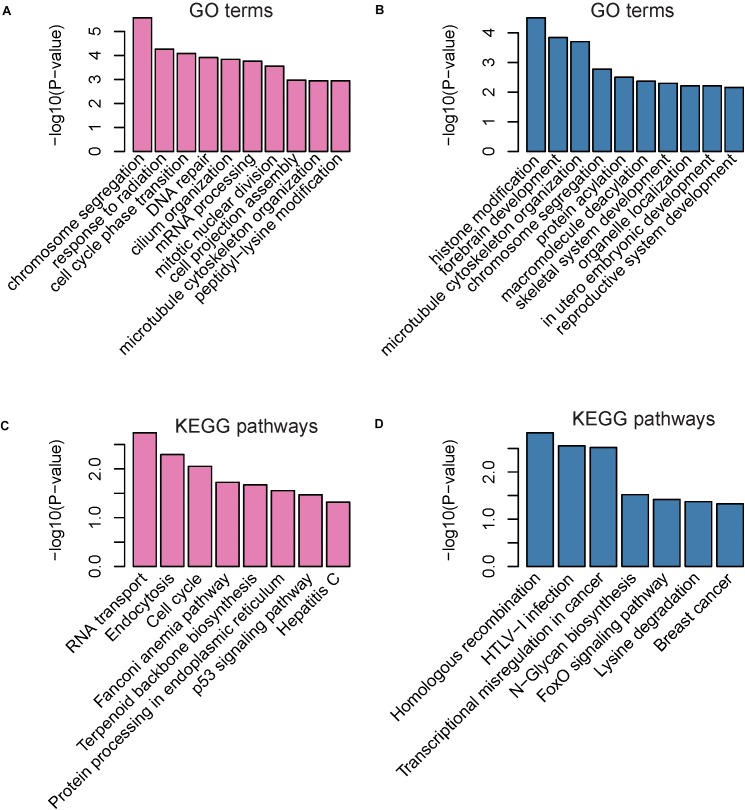
Gene Ontology (GO) and Kyoto Encyclopedia of Genes and Genomes (KEGG) enrichment of parental genes of dysregulated circRNAs The enriched GO terms and KEGG pathways are presented in **(A–D)**. The pink and blue bars represent the GO terms or KEGG pathways enriched by parental genes of up-regulated and down-regulated circRNAs, respectively.

Gene ontology analysis indicated that the upregulated genes were mainly involved in the regulation of chromosome segregation, response to radiation, cell cycle phase transition, DNA repair, cilium organization, mRNA processing, mitotic nuclear division, cell projection assembly, microtubule cytoskeleton organization, and peptidyl-lysine modification (Figure 3A). Furthermore, the downregulated genes were mainly enriched in categories associated with the regulation of histone modification, forebrain development, microtubule cytoskeleton organization, chromosome segregation, protein acylation, macromolecule deacylation, skeletal system development, organelle localization, in utero embryonic development, and reproductive system development (Figure 3B). These up-regulated pathways noted above indicated that the up-regulated parental genes may participate in the process of DNA damage under oxidative stress.

Kyoto encyclopedia of genes and genomes pathway analysis revealed that upregulated genes were primarily enriched in pathways associated with RNA transport, endocytosis, cell cycle, fanconi anemia pathway, terpenoid backbone biosynthesis, protein processing in endoplasmic reticulum, p53 signaling pathway, and hepatitis C (Figure 3C). In accordance with the enriched GO terms, the up-regulated genes were significantly enriched in pathways related to DNA damage under oxidative stress. Downregulated genes were mainly associated with homologous recombination, HTLV-I infection, transcriptional misregulation in cancer, N-Glycan biosynthesis, FoxO signaling pathway, lysine degradation, and breast cancer (Figure 3D).

### Alternative Circularization of Dysregulated circRNAs in Exonic Regions

**FIGURE 4:**
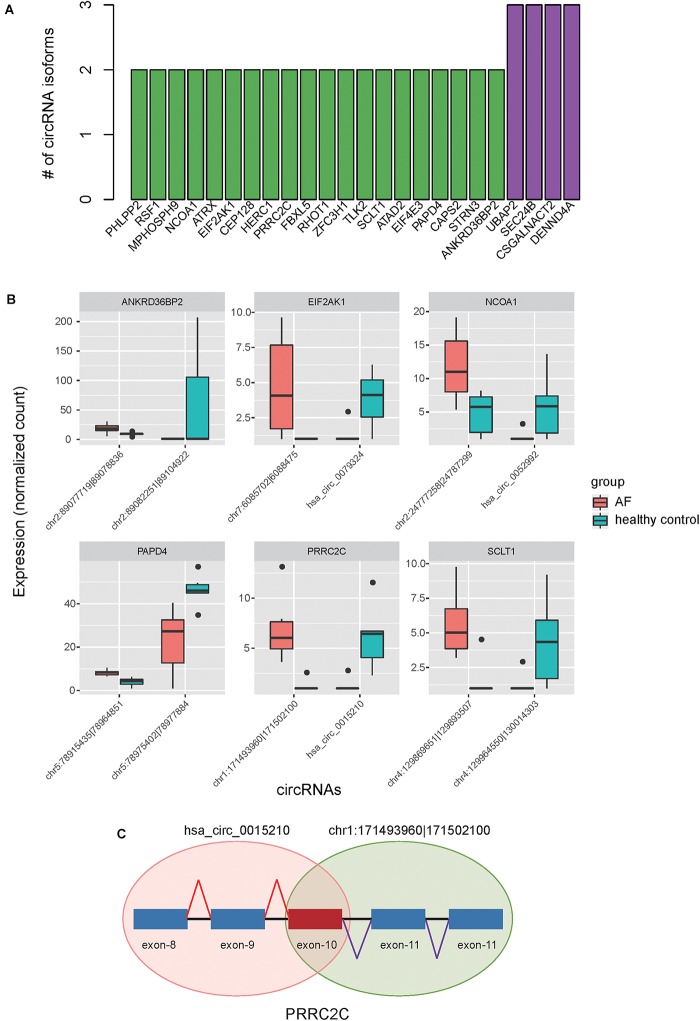
Alternative circularization of the dysregulated circRNAs. **(A)** The number of circRNA isoforms for the parental genes with alternative circularization. **(B)** Expression levels of switched circular RNA isoforms for six parental genes between AF and healthy controls. **(C)** The schematic diagram for the two circRNA isoforms in PRRC2C with differential usage of the 10-th exon.

Alternative RNA circularization was determined only by back-splicing sites, and therefore we inferred the gene structure of circRNAs based on annotated transcripts. To avoid the occurrence of fuzzy gene structure, only exonic circRNAs were included in such analyses. We found that 24 genes had more than two circRNA isoforms, of which, 20 produced two isoforms, and 4 produced three isoforms (Figure 4A). Interestingly, we also observed that six genes, including NCOA1, ANKRD36BP2, PAPD4, PRRC2C, SCLT1, and EIF2AK1, produced circRNA isoforms with opposite expression patterns (Figure 4B), indicating that these expression-switched circRNA isoforms may have opposite functions. Moreover, the expression-switched circRNA isoforms for 5 of 6 genes did not have overlapping exons. Exceptionally, the two circRNA isoforms, hsa_circ_0015210 and chr1:171493960-171502100, produced by PRRC2C, shared the 10-th exon (Figure 4C), indicating that the differential usage of the 10-th exon was associated with AF.

### Functional Annotation of circRNAs by Integrating Potential ceRNA and PPI Networks

To further investigate the regulatory mechanism of circRNAs, we predicted the miRNA binding sites for each circRNA. Finally, we predicted 43,307 miRNA-circRNA interactions by Miranda v3.3a with a strict mode. As circRNAs could also act as ceRNAs by competing for miRNAs with mRNAs, we also collected 322,389 experimentally validated miRNA-mRNA interactions from MiRTarBase (Chou et al., 2018), of which, 12,930 were miRNA/dysregulated mRNA interactions.

**FIGURE 5:**
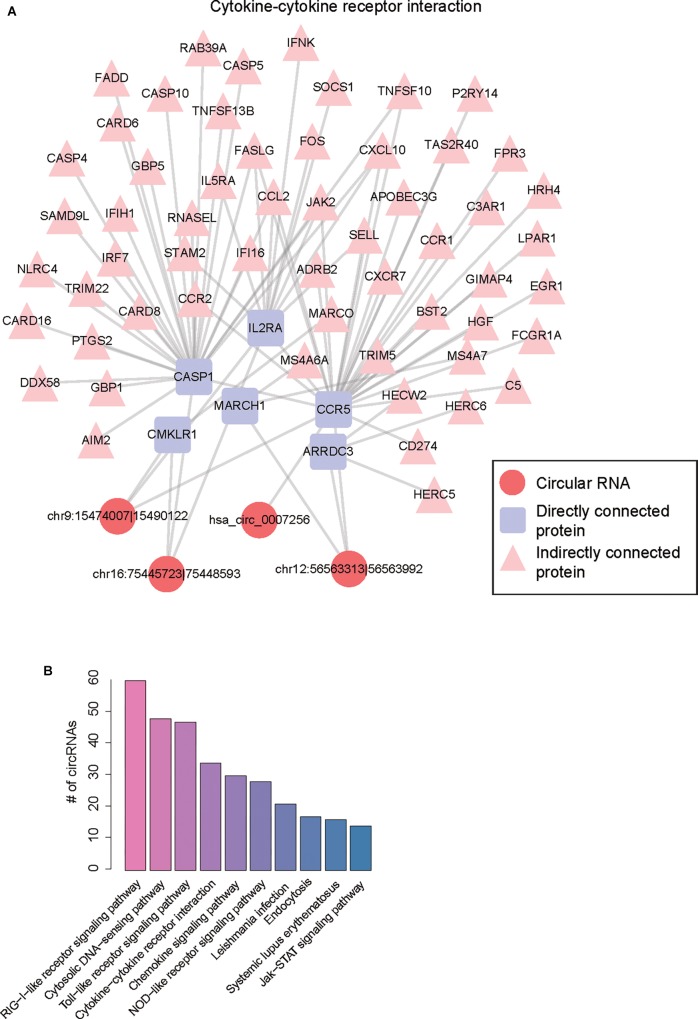
Functional annotation of dysregulated circRNAs by merging ceRNA and PPI network. **(A)** The merged network involving protein-protein and circRNA-mRNA interactions. **(B)** The number of circRNAs for the top-ten most frequently enriched pathways.

To construct the ceRNA network, we estimated the significance of shared miRNAs for each circRNA-mRNA pair. We predicted 1,025 up-regulated and 245 down-regulated circRNA-mRNA pairs by one-tailed Fisher’s exact test (*P*-value < 0.0001), involving 246 dysregulated circRNAs. Furthermore, we also mapped the up-regulated and down-regulated protein-coding genes to PPI network, respectively. To characterize the biological functions of circRNAs, we merged the potential ceRNA network with the PPI network (Supplementary Table S2), and performed KEGG enrichment analysis on the genes connected to the circRNAs within one node in the merged network. Finally, 130 of the 246 dysregulated circRNAs in the merged network were successfully characterized by more than one pathway. Notably, the five circRNAs, including chr9:15474007-15490122, chr16:75445723-75448593, hsa_circ_0007256, chr12:56563313-56563992, and hsa_circ_0003533, showed the highest significance in the enrichment analysis, and four of them were enriched in cytokine-cytokine receptor interaction (Table 1 and Figure 5A). Notably, CCR5, which acted as a receptor for chemokines, was the target of three circRNAs in the ceRNA network, suggesting that the three circRNAs may enhance the activity of cytokine-cytokine receptor interaction through CCR5. As shown in Figure 5B, the pathways charactering top-ten number of circRNAs, such as RIG-I-like receptor signaling pathway, Toll-like receptor signaling pathway, NOD-like receptor signaling pathway, and JAK-STAT signaling pathway, were mostly related to inflammation, suggesting that the circRNAs enriched in these pathways may participate in biological processes of inflammatory response (Supplementary Table S3).

**Table 1:** The top-five circRNAs with the highest significance level by KEGG enrichment analysis.

circRNA	KEGG pathway	*P*-value	Genes
chr9:15474007-15490122	Cytokine-cytokine receptor interaction	4.97E-18	IL2RA,CCR5,CXCL10,CCR1,FASLG,CCL2,CCR2,IL5RA,IFNK,HGF,TNFSF10
chr16:75445723-75448593	Cytokine-cytokine receptor interaction	1.79E-15	IL2RA,IL5RA,IFNK,TNFSF10,FASLG,CCL2,CXCL10,CCR5,CCR2,TNFSF13B
hsa_circ_0007256	Cytokine-cytokine receptor interaction	4.09E-15	CCR5,CXCL10,CCR1,FASLG,IL2RA,CCL2,CCR2,HGF,TNFSF10
chr12:56563313-56563992	Cytokine-cytokine receptor interaction	4.44E-14	CCR5,CXCL10,CCR1,FASLG,IL2RA,CCL2,CCR2,HGF,TNFSF10
hsa_circ_0003533	RIG-I like receptor signaling pathway	4.58E-14	CXCL10,IRF7,DDX58,ISG15,FADD,CASP10,IFIH1,DHX58


## Discussion

Circular RNAs are an emerging class of RNA species that may play a critical regulatory role in gene expression control. CircRNAs can serve as diagnostic biomarkers for many diseases (Han et al., 2018) due to their abundant, stable, and cell- or tissue-specific expression (Bachmayr-Heyda et al., 2015; Li et al., 2018). However, the association between circRNAs and AF is still not clear.

In this study, we used RNA sequencing data to identify and quantify the circRNAs. Differential expression analysis of the circRNAs identified 250 up- and 126 down-regulated circRNAs in atrial fibrillation patients compared with healthy donors, respectively (Figure 2A,B). The hierarchical clustering analysis of the dysregulated circRNA expression profiles revealed that the samples with AF could be clearly distinguished from the healthy donors (Figure 2C), suggesting that the dysregulated circRNAs may act as potential AF diagnostic biomarkers in lymphocytes. GO and KEGG analysis of the parental genes of the dysregulated circRNAs indicated that parental genes of dysregulated circRNAs may participate in the process of DNA damage under oxidative stress (Figure 3A,C). The down-regulated parental genes were mainly associated with homologous recombination, HTLV-I infection, transcriptional misregulation in cancer, N-Glycan biosynthesis, FoxO signaling pathway, lysine degradation, and breast cancer (Figure 3B,D). To examine whether circRNA isoforms originated from the same genes were dysregulated in AF, we inferred the gene structure of circRNAs based on annotated transcripts. Interestingly, among the dysregulated circRNA isoforms, six genes, including NCOA1, ANKRD36BP2, PAPD4, PRRC2C, SCLT1, and EIF2AK1, were identified to produce circRNA isoforms with opposite expression patterns, indicating that these expression-switched circRNA isoforms may have opposite functions (Figure 4B). Notably, the two circRNA isoforms, hsa_circ_0015210 and chr1:171493960-171502100, produced by PRRC2C, shared the 10-th exon (Figure 4C), indicating that the differential usage of the 10-th exon was associated with AF. To further annotate the dysregulated circRNAs, we constructed and merged the ceRNA network and PPI network. In the merged network, 130 of 246 dysregulated circRNAs were successfully characterized by at least one pathway. Notably, the five circRNAs, including chr9:15474007-15490122, chr16:75445723-75448593, hsa_circ_0007256, chr12:56563313-56563992, and hsa_circ_0003533, showed the highest significance in the enrichment analysis, and four of them were enriched in cytokine-cytokine receptor interaction (Table 1). In summary, these dysregulated circRNAs may participate in biological processes of inflammatory response.

In this study, there also existed some limitations. Firstly, more samples were needed considering the small sample size in the present study. Secondly, we provided a set of dysregulated circRNAs associated with AF, however, further experimental validation would be required for future verification. Moreover, specific functions of those dysregulated circRNAs had not been further excavated in this study. We hope to conduct further researches with a larger samples group, to perform experimental validation and much deeper analysis in the near future.

## Conclusion

We identified six genes, including NCOA1, ANKRD36BP2, PAPD4, PRRC2C, SCLT1 and EIF2AK1, producing circRNA isoforms with opposite expression patterns, and characterized some inflammation-related circRNAs, such as chr9:15474007-15490122, chr16:75445723-75448593, hsa_circ_0007256, chr12:56563313-56563992, and hsa_circ_0003533, which may be associated with inflammatory responses in AF. To our knowledge, this is the first study to uncover the association between circRNAs and AF, which not only improves our understanding of the roles of circRNAs in AF, but also provides candidates of potentially functional circRNAs for AF researchers.

## Author Contributions

XL led the research team. XL and XH conceived and designed the study. LC and SW developed the methodology. KX and WJ collected the sample. MQ and YZ analyzed and interpreted the data. XH wrote, reviewed, and revised the manuscript.

## Conflict of Interest Statement

The authors declare that the research was conducted in the absence of any commercial or financial relationships that could be construed as a potential conflict of interest.

## Footnotes

1 https://www.ncbi.nlm.nih.gov/sra

2 http://www.ncbi.nlm.nih.gov/sra/docs/toolkitsoft/

3 http://www.genome.ucsc.edu

4 https://string-db.org

5 http://www.cytoscape.org

6 http://www.r-project.org/

7 https://www.ncbi.nlm.nih.gov/sra

8 http://www.circbase.org/

## Supplementary Material

Table S1 A gene set enrichment analysis of their parental genes based on biological processes from GO and pathways from KEGG database.

Table S2 The potential ceRNA network merged with the PPI network of circRNAs.

Table S3 The KEGG pathways involving 102 circRNAs.
